# Multimodal graph learning for chagas disease classification

**DOI:** 10.1007/s11517-026-03631-y

**Published:** 2026-07-17

**Authors:** Gabriel Carcedo-Rodríguez, Erik Molino-Minero-Re, Jorge Perez-Gonzalez, Nidiyare Hevia-Montiel

**Affiliations:** 1 Master Program in Computer Science and Engineering, Universidad Nacional Autónoma de México, Mérida, Yucatán México; 2 Unidad Académica del Instituto de Investigaciones en Matemáticas Aplicadas y en Sistemas, Universidad Nacional Autónoma de México, Tablaje Catastral No 6998, Mérida, 97357 Yucatán México

**Keywords:** Multimodal learning, Graph neural network, Graph attention network, Variational Graph Autoencoder, Chagas disease, Interpretability, High dimensionality

## Abstract

**Abstract:**

The accurate classification of Chagas disease stages based on *Trypanosoma cruzi* infection in experimental models faces challenges due to the scarcity of data and the high dimensionality of diagnostic sources. This study proposes an architecture based on Graph Attention Networks on fully connected graphs to combine multimodal features (Electrocardiogram, Echocardiogram, Doppler, and ELISA). A Variational Graph Autoencoder was used to implement a data augmentation strategy that reduces overfitting and optimizes generalization, producing synthetic subjects with a biological covariance structure. The results demonstrate that the proposed methodology is effective compared to state-of-the-art classifiers, achieving an AUROC of 100% in the identification of infection stage through multimodal fusion and significantly optimizing performance in complex modalities such as Doppler. The pathophysiological consistency of the model’s decisions was also validated using an interpretability scheme (GNNExplainer), which allowed the detection of important clinical features and established GATs as a robust method in the classification of *Trypanosoma cruzi* infection stages.

**Graphical abstract:**

**Figure:**
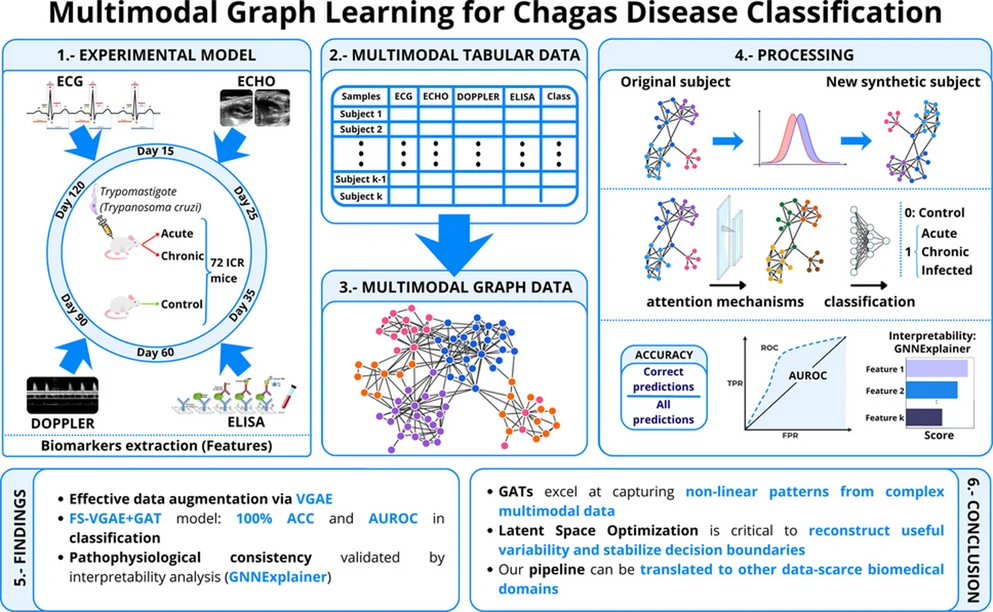
Scheme of the proposed multimodal graph learning framework for Chagas disease classification. It presents the core processing stages, including graph data modeling, synthetic data augmentation, and attention-based classification

## Introduction

The parasitic illness with the greatest impact in Latin America is Chagas disease, caused by the protozoan *Trypanosoma cruzi*, which is endemic in 21 countries in this region. It occurs in two stages: the acute stage in the first months of infection, characterized by high parasitemia, and the chronic stage, where heart failure and arrhythmias begin to appear. According to reports from the World Health Organization (WHO) [18] and the Pan American Health Organization (PAHO) [14], globally, more than 7 million people are infected and more than 100 million are at risk of contracting it, with an annual average of 10,000 deaths. Between 2018 and 2024, 6,498 cases were documented in Mexico; of these, more than 90% correspond to the chronic stage, in which permanent lesions and damage are already evident.

A key challenge is detecting infection in its acute phase, as it is often asymptomatic, complicating early diagnosis and increasing the risk of serious heart damage or sudden death in advanced stages. Current diagnostic methods are based on functional studies, including electrocardiogram (ECG), echocardiography (ECHO), and spectral Doppler ultrasound, and primarily, serological exams such as enzyme-linked immunosorbent assay (ELISA). However, in computational studies of Chagas disease, due to limited access to human data, available sample sizes are frequently restricted to critically small cohorts due to the inherent difficulties of data collection [17]. Consequently, the use of experimental murine models is crucial, as it provides a controlled environment to capture the evolution of cardiac biomarkers and generate robust databases that allow the evaluation of noninvasive diagnostic strategies and the characterization of the progression of infection, overcoming the scarcity of clinical data [4]. In recent years, machine learning (ML) has established itself as a powerful tool for predicting and classifying biomedical issues, as it enables the extraction of complex patterns from biomedical data [15].

Several studies analyzing Chagas disease have used ML to classify infection stages in murine models. By applying algorithms such as Random Forest (RF) and Support Vector Machines (SVM) to ECG signals, Haro et al. [6] achieved a multi-class classification accuracy of up to 91.3%. As a complement, Vazquez et al. [17] proposed a generative model based on Weighted Variational Autoencoders (W-VAE) to augment and impute multimodal data (Doppler, ECHO, ELISA, and ECG), achieving 95% accuracy despite high levels of missing data.

Hevia-Montiel et al. [8] presented an approach to automatically classify *T. cruzi* infection stages in a murine experimental model, using ML approaches on the multimodal dataset used in this research, including ECG, ECHO, DOPPLER signals and ELISA results. They used traditional classifiers such as RF, SVM, Decision Trees (DT), and Extra Trees (ETC), obtained competitive metrics for accuracy (ACC) and area under the ROC curve (AUROC), and achieved completely accurate classifications in binary situations with serological information. In this research, these experiments are repeated using a Graph Neural Network (GNN) architecture, which allows for a direct comparison of performances and shows remarkable advances in terms of robustness and accuracy of our proposed approach compared to conventional methods.

Furthermore, Deep Learning (DL) techniques have shown significant progress in disease prediction using complex biomedical data. In this field, Kipf and Welling [12] introduced Graph Convolutional Networks (GCN), proposing an effective method to efficiently approximate spectral convolutions on graphs. To overcome the GCN’s structural limitation of assigning weights to node neighborhoods based solely on the topological structure of the graph, Veličković et al. [16] introduced Graph Attention Networks (GAT), which include masked self-attention mechanisms. Unlike GCNs, GATs allow for the dynamic assignment of importance coefficients to each neighbor. Thus, the network can learn to value the connections that are most important for the classification task without requiring an established graph structure. Additionally, Brody et al. [3] proposed a dynamic attention mechanism (GATv2) that improves the model’s capability to capture complex structural relationships between clinical entities. In the interpretability field, for causal explanations, Ying et al. [20] proposed GNNExplainer, a model-agnostic approach that identifies compact subgraphs and subsets of node features that play a crucial role in the GNN prediction.

The implementation of the GAT framework is a key component in addressing the inherent challenges of limited data; by incorporating graph topology that constrains the optimization space [5]. This capability is critical for processing biomedical multimodal variables. As validated by Margeloiu et al. [13] and Hossain et al. [9], graph-based learning strategies are effective in high-dimensional and limited-sample environments, allowing the attention mechanism to prioritize relevant clinical biomarkers while maintaining stable generalization. Aligning with these biomedical advantages, Xuan et al. [19] presented GCNLDA, a combined model that links GCNs and convolutional networks, incorporating attention mechanisms to deduce connections between diseases and long noncoding RNAs (lncRNAs) from a diverse network of biological nodes.

However, the generalization of these models often depends on the availability of labeled data, a common issue in biomedical research. To mitigate this limitation and avoid model overfitting, data augmentation strategies become highly relevant, especially those based on generative approaches. Kingma and Welling [10] established the theoretical foundations of the Variational Autoencoder (VAE), suggesting a stochastic variational inference framework and a reparameterization technique that facilitate the learning of continuous latent distributions for the effective creation of new synthetic samples. Extending this to the non-Euclidean domain, Kipf and Welling [11] created the Variational Graph Autoencoder (VGAE). This model uses a GCN encoder to capture the structure and features of the graph in a probabilistic latent space, employing an inner product decoder to reconstruct or create new instances. In biomedicine, VGAs have the ability to capture underlying topological distributions, preserving the original statistical characteristics of the data and improving the training set.

This research contributes a comprehensive methodological framework for the binary classification of *Trypanosoma cruzi* infection stages in multimodal experimental models, addressing the inherent difficulties caused by the lack and high dimensionality of biomedical data. An architecture based on Graph Attention Networks (GAT) on fully connected graphs is suggested, which allows overcoming the restrictions of conventional classifiers that assume independence between characteristics. This perspective enables the capture of nonlinear dynamic interactions between multimodal parameters (ECG, ECHO, Doppler, ELISA), which significantly improves sensitivity in detecting the early (acute) and late (chronic) disease stages. It also shows that a Variational Graph Autoencoder (VGAE) is effective in generating synthetic subjects that maintain the biological covariance structure. The incorporation of these data served as an essential regularizer, reducing overfitting in situations of insufficient samples and increasing the model’s ability to generalize in complex contexts such as multimodal fusion. In addition, a GNNExplainer-based interpretability module is used that goes beyond “black box” prediction, corroborating the results that the model prioritizes logical pathophysiological features. Finally, it was determined that feature selection through voting prior to data augmentation using VGAE, together with the graph-based DL model for classification (GAT), is an effective way to integrate heterogeneous sources. This allows for higher classification metrics in tasks where traditional methods proved unstable. These contributions make interpretable and generative GNNs effective tools for accurate classification in experimental cardiovascular research.

## Methods

**Fig. 1:**
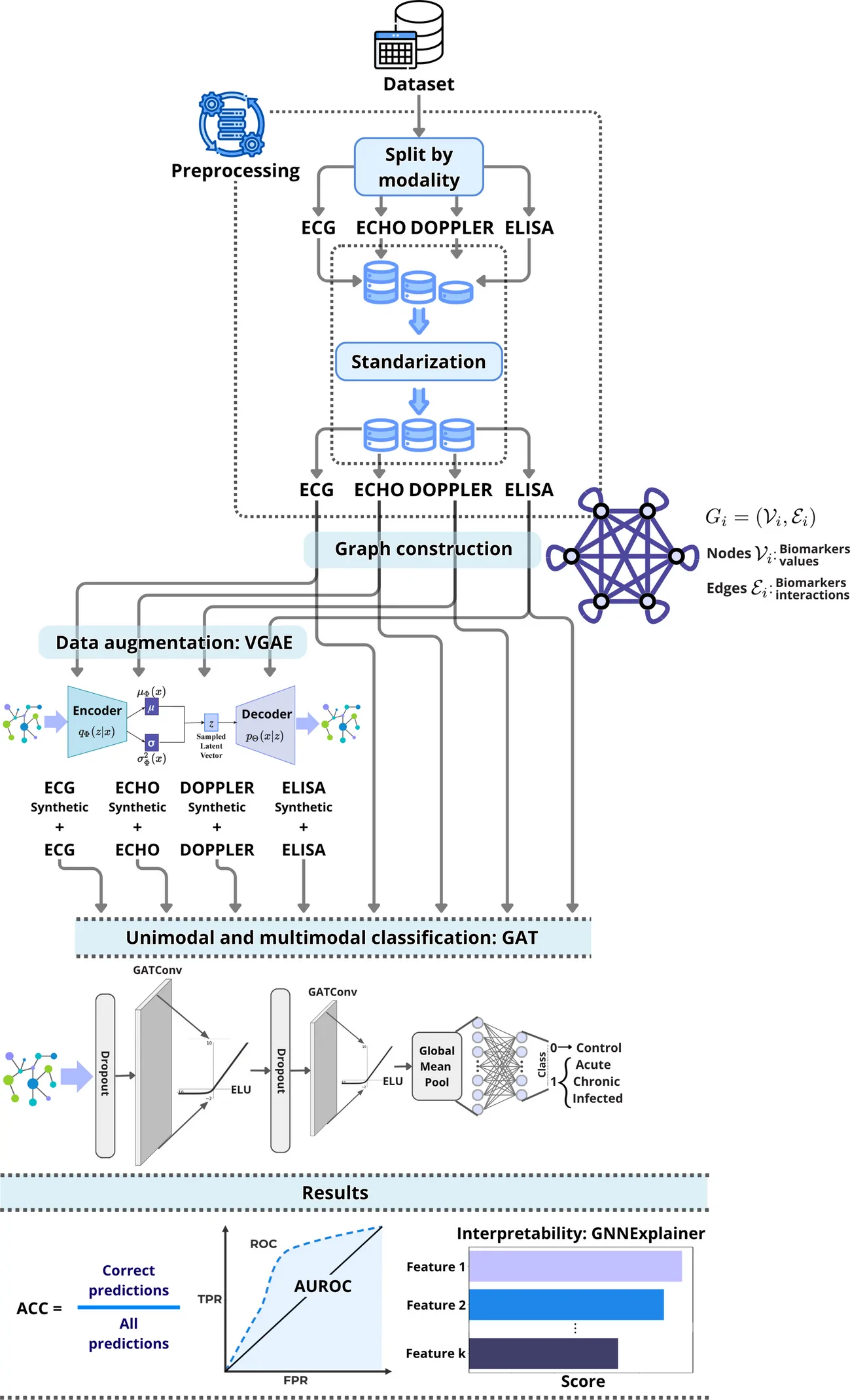
General pipeline of the methodology used to classify *T. cruzi* infection stages in experimental models

Figure 1 illustrates the pipeline developed to classify murine models infected with *T. cruzi*. The biomarkers obtained by ECG, ECHO, Doppler, and ELISA were analyzed and preprocessed, evaluating their performance by modality and as a whole, in a multimodal manner. To this end, each modality was standardized in isolation, fully connected graphs were constructed for each subject, with nodes representing biomarkers, and a VGAE model was used to generate synthetic data for each modality. The results of the proposed GAT model were compared before and after data augmentation and with those obtained by ML algorithms from the study by Hevia-Montiel et al. [8], using the accuracy (ACC) and area under the ROC curve (AUROC) metrics.

To ensure statistical robustness and the possibility of model generalization given the inherent limitations of data, the experimental design incorporates a strict stratified cross-validation scheme with *k* folds (*k*-fold cross-validation). Unlike approaches that assume independence between characteristics, the suggested architecture models each subject as a unique relational structure; therefore, the workflow is not reduced to maximizing performance metrics, but prioritizes the extraction of biological knowledge. This is achieved by adding an interpretability module that enables the analysis of model decisions, ensuring that the binary classification of infection stages (control vs. acute/chronic/general infection) is based on logical physiological patterns and not on stochastic artifacts present in the dataset. The characteristics of the study population and the specific protocols for each step of the investigation are detailed below.

### Experimental setup

The data acquisition was based on a controlled murine model of *Trypanosoma cruzi* infection, conducted in strict accordance with ethical guidelines and approved by the corresponding institutional committee (CIRB-006-2017) [8]. A total of 72 female ICR mice were utilized to model both acute (sampled at 15, 25, and 35 days post-infection) and chronic (sampled at 60, 90, and 120 days) disease stages. For each phase, subjects were equally divided into control and infected groups, with the progression of the infection continuously validated by peripheral blood parasite counts.

### Dataset

Derived from the experimental model, diagnostic variables were extracted utilizing four multimodal techniques: ECG, ECHO, Doppler, and ELISA. This yielded a perfectly balanced dataset comprising 72 subjects distributed into four distinct subclasses of 18 subjects each: control for acute stage, acute infected, control for chronic stage, and chronic infected. To rank diagnostic relevance, the criteria established by Hevia-Montiel et al. [8] were adopted, who proposed selection schemes based on voting and empirical knowledge. Table 6 details the final dataset, which contains 72 samples and 67 variables: 5 from ECHO, 14 from ECG, 45 from Doppler, and 3 from ELISA.

The input variables show significant dimensional heterogeneity due to the multimodal nature of the dataset, including very different physiological units (e.g., millivolts and milliseconds in ECG, cm/s in Doppler, and optical density in ELISA). A stratified standardization protocol was established for each modality to reduce the numerical bias that scale disparities could generate in the attention mechanisms.

Specifically, before graph construction or data fusion, a Z-score transformation [2] was applied independently to each feature matrix per modality $M \in \{$ECG, ECHO, Doppler, ELISA*\}*. This approach ensures that each feature is centered at 0 with a unit standard deviation, maintaining the relative biological variance between subjects while suppressing the impact of absolute magnitudes, ensuring attention coefficients based on the statistical relevance of the biomarker for classification, rather than on the scale of its measurement unit.

Each subject *i* was modeled as an undirected graph $G_i = (\mathcal {V}_i, \mathcal {E}_i)$ to represent the functional relationships between physiological parameters. The set of nodes $\mathcal {V}_i$ refers to the biomarkers that have been extracted. The set of edges $\mathcal {E}_i$ models the interactions between these nodes (biomarkers). Each node encapsulates the standardized value in numerical terms of each of these features. A fully connected topology ($K_n$) was chosen, where each node is connected to all other nodes in the graph, including a loop or self-loop to itself. Independent graphs were generated for every modality (ECG, ECHO, Doppler, ELISA), as well as integrated multimodal graphs.

The choice of a fully connected structure is based on the ability of the subsequent attention mechanism (GAT) to perform dynamic data filtering. This configuration enables the neural network to explore the entire space of possible interactions between biomarkers, rather than establishing a static adjacency matrix based on arbitrary correlation limits, which could leave out significant nonlinear interactions. Thus, the model learns to assign near-zero weights to irrelevant connections and to strengthen those that are of high clinical importance for infection stage classification.

### Data augmentation

As discussed in the state of the art, the scarcity of labeled biological samples (Small Data) represents a significant obstacle to the robust training of models such as GATs. This increases the potential for overfitting and reduces generalization ability. A Variational Graph Autoencoder (VGAE) was designed and trained to generate synthetic subjects that follow the biological topology of each diagnostic modality.

**Fig. 2:**
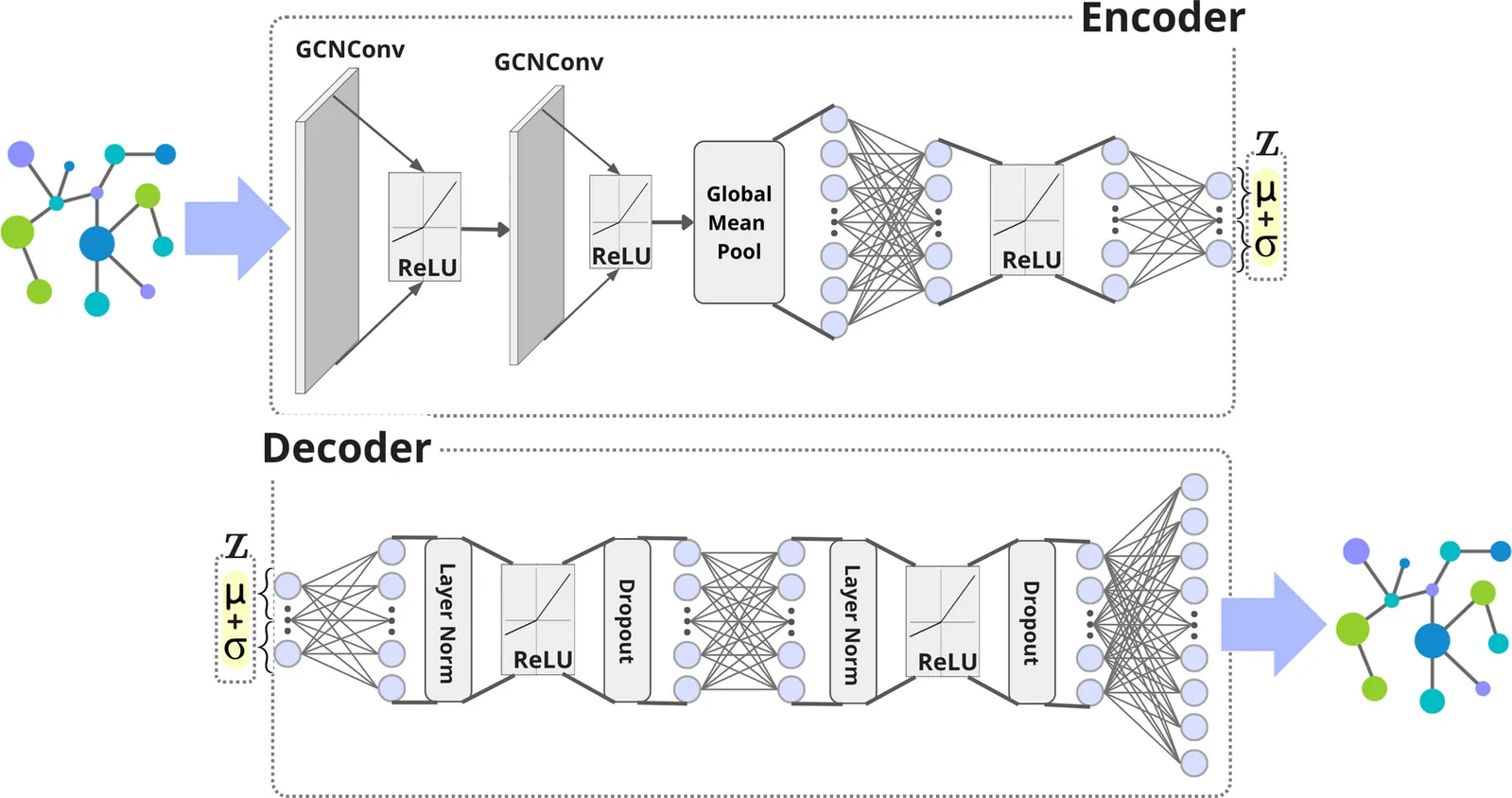
Variational Graph Autoencoder (VGAE) model architecture proposed for synthetic data augmentation

The proposed architecture, illustrated in Fig. 2, consists of two main components: a GCN-based probabilistic encoder and a multilayer perceptron (MLP) decoder to compose the VGAE model. The encoder takes as input the patient feature matrix *X* and the adjacency matrix *A*. It uses two Convolutional Graph layers followed by a Global Mean Pooling operation to compress the information from all nodes into a compact latent representation of the entire graph. Unlike a traditional autoencoder that maps the input to a fixed point, this encoder predicts the parameters of a multivariate Gaussian distribution in the latent space: the mean vector *μ * and the logarithm of the standard deviation $\log (\sigma )$.

Variational inference is defined below by Eq. 1:

1 $$\begin{aligned} q(z | X, A) = \mathcal {N} \left( z | \mu , \text {diag} \left( \sigma ^2\right) \right) \end{aligned}$$

where *z* is the latent vector sampled using the reparameterization trick:

2 $$\begin{aligned} z = \mu + \sigma \odot \epsilon , \quad \epsilon \sim \mathcal {N}(0, I) \end{aligned}$$

The Decoder is a dense neural network (MLP) with Layer Normalization and Dropout regularization. Its task is to reverse the compression process to create new synthetic biomarker values, reconstructing the patient’s original feature vector $\hat{X}$ from the sampled latent space *z*.

The training of the generative models was performed in a first strategy that was independent and stratified for each diagnostic modality ($M \in \{$ECG, ECHO, Doppler, ELISA*\}*) and clinical class ($C \in \{$Control, Acute, Chronic*\}*). For each configuration (*M*, *C*), the data from training set were used as the starting point for the generative model. Meanwhile, the test set was kept completely uncompromised, preserving entirely real subjects strictly for final performance evaluation. The training setup is explained in Table 7. The loss function used in training consists of the following two terms with the aim of maximizing the variational lower bound:

1. **Reconstruction loss (** $\mathcal {L}_{rec}$ **):** Obtained through the MSE between the original features *X* and the reconstructed features $\hat{X}$, forcing the model to generate data that represent physiological reality.
2. **Kullback-Leibler divergence (KL) (** $\mathcal {L}_{KL}$ **):** This acts as a regularizer that ensures a fluid and continuous latent space, suitable for generation, by penalizing the discrepancy between the learned latent distribution *q*(*z*|*X*) and a standard Gaussian prior distribution $\mathcal {N}(0, I)$.

The complete loss function is defined as:

3 $$\begin{aligned} \mathcal {L} = \mathcal {L}_{rec} + \beta \cdot \mathcal {L}_{KL} \end{aligned}$$

where *β * is a tunable weight hyperparameter [7], that was gradually increased during training (warm-up) to stabilize convergence from 0 to 0.1, until 10% of standard KL penalty, as a final fine-tuning mechanism during the last training epochs, adjusting the synthetic data distribution without overpowering the reconstruction.

Once the convergence of the models had stabilized, the generation (inference) phase was carried out. For each of the four clinical subclass (originally consisted of 18 subjects each), a strict partitioning was maintained: 15 subjects were allocated to the training set, and 3 were reserved exclusively for testing. Latent vectors from the learned distribution $z \sim \mathcal {N}(\mu , \sigma )$ were then sampled until 15 new synthetic subjects per subclass were generated, establishing a 1:1 augmentation ratio relative to the training partition. This procedure resulted in a total of 132 subjects (72 real and 60 synthetic), by corresponding 33 subjects per subclass (15 real training, 15 synthetic training, and 3 real testing). Finally, the probability densities (KDE) of each synthetic biomarker were compared with the original real data to verify the quality of the generated data.

In order to evaluate whether dimensionality reduction focused on biological relevance improves the quality of data generation and, consequently, classification, a second data augmentation strategy was implemented. Unlike the general approach described above, where VGAE learns the joint distribution of all available features, the FS-VGAE model was trained using only the subsets of features identified as discriminating for each modality in the Feature Selection (FS) algorithms reported by Hevia-Montiel et al. [8] based on the voting selection scheme (detailed in Table 6). The premise is that, by restricting the autoencoder input space to biomarkers with greater statistical weight, FS-VGAE is forced to model the latent structure of essential pathological variability, eliminating noise introduced by redundant or low-ranking features.

### Classification of *T. cruzi* infection stages

To classify the different stages of *T. cruzi* infection, a framework based on Graph Attention Networks (GAT) was developed. This section details the proposed architecture, the training setup designed to mitigate data scarcity, and the experimental scenarios established to evaluate unimodal and multimodal diagnostic approaches.

**Fig. 3:**
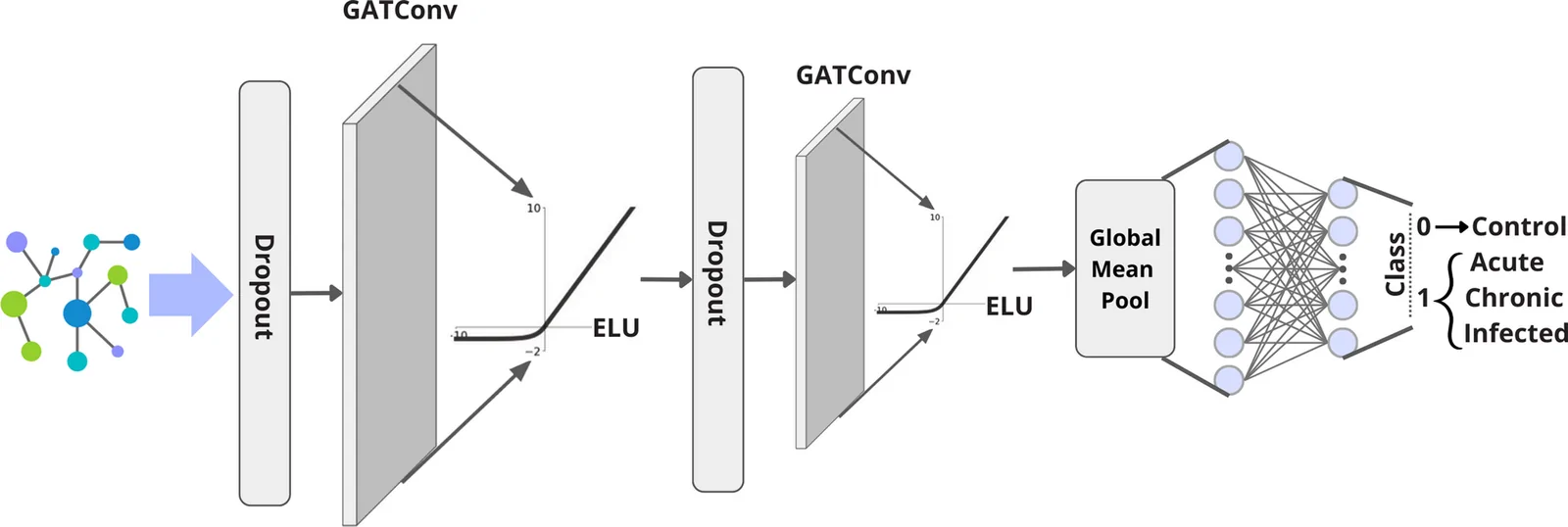
Proposed Graph Attention Network (GAT) model architecture, illustrating the flow of information from input nodes to final classification

An architecture based on Graph Attention Networks (GAT) was designed and implemented to classify *T. cruzi* infection stages (see Fig. 3). The proposed model applies self-attention mechanisms to dynamically evaluate the relevance of interactions between features (biomarkers).

Our model consists of two sequential attention layers. The first layer uses a multi-head attention mechanism with *K* independent heads, allowing different representation subspaces to be learned simultaneously. Formally, for a pair of nodes (*i*, *j*), the attention coefficient $a_{ij}$ is calculated using the variant GATv2 [3]:

4 $$\begin{aligned} a_{ij} = a^T \text {LeakyReLU}\left( W \cdot [h_i \Vert h_j] \right) \end{aligned}$$

where *W* is the learnable weight matrix, *a* is the attention vector, *h* are the nodal features, and *‖ * denotes concatenation. The normalized coefficients $\alpha _{ij}$ are obtained by applying the Softmax function to $a_{ij}$.

To mitigate the gradient vanishing problem in standardized data, the ReLU activation function was replaced by the Exponential Linear Unit (ELU), ensuring that information propagates even when biomarkers have negative values (Z-scores < 0). The architecture ends with a Global Mean Pooling layer, which concentrates the information from the entire graph into a single embedding vector and then includes a linear classification layer. The model was trained through supervised learning, improving the network weights to minimize the cross-entropy loss function. The loss for a group of *N* subjects is defined, considering that the classification tasks are binary (control vs. acute/chronic/general infection), as follows:

5 $$\begin{aligned} \mathcal {L} = - \frac{1}{N} \sum _{i=1}^{N} \left[ y_i \log (\hat{p}_i) + (1 - y_i) \log (1 - \hat{p}_i) \right] \end{aligned}$$

where $y_i$ is the actual label (0: control, 1: acute/chronic/general infection) and $\hat{p}_i$ is the probability predicted by the model after applying the Softmax function. The setup for training is described in Table 7.

To evaluate the effectiveness of our proposed approach and establish a direct comparison with the state of the art, we replicated the experimental scenarios defined by Hevia-Montiel et al. [8] using the evaluation metrics described in Appendix C. Three binary classification schemes were designed: (a) control vs. acute to early detection of infection, (b) control vs. chronic to late-stage identification, and (c) control vs. general i infection for overall classification of healthy subjects versus infected subjects (irrespective of stage). Each of these schemes was carried out using two approaches to data usage: unimodal with training independent GAT models for each data modality (ECG, ECHO, Doppler, ELISA) and multimodal integrating features from multiple modalities. For multimodal fusion, three feature selection strategies were compared: (i) all available variables, (ii) empirical selection based on expert knowledge, and (iii) voting selection based on statistical relevance reported in [8]. Model performance was measured using ACC and AUROC, reporting the mean and standard deviation for cross-validation, as well as the final test results.

### Interpretability: *Post Hoc* analysis

To review the decisions of the model and prevent the “black box” effect, an interpretability module was included, considering its crucial importance in medical diagnosis. This method makes it possible to check whether the classification is based on spurious correlations or consistent physiological indicators.

To measure the individual contribution of each biomarker to the final decision, GNNExplainer [20], a model-agnostic interpretation tool, was used. This technique aims to determine the subgraph $G_S \subseteq G$ and the attribute mask $X_S$ that maximizes mutual information with the model prediction $\hat{y}$. Mathematically, GNNExplainer optimizes a soft mask on the nodes (biomarkers), learning an importance value *∈ [0, 1]* for each variable. This approach addresses counterfactual causality, identifying which perturbations in biomarkers would change the diagnosis of the infection stage. This enables the creation of an individual clinical importance ranking for each subject, allowing validation of whether the decisive biomarkers are in line with what has been published in the medical literature on Chagas disease derived from *T. cruzi* infection.

## Results

This section presents the results obtained in each methodological section. The performance of GAT architecture is reported in the three defined binary classification schemes: (a) control vs. acute, (b) control vs. chronic, and (c) control vs. general infection. The analysis classifies the results according to the individual diagnostic method and fusion strategies, as well as calculating the improvement in performance due to the increase in synthetic data through VGAE. In addition, the biological consistency of the predictions is analyzed employing GNNExplainer.

### Data augmentation

Table 1 presents the convergence metrics for generative models in two schemes: using the complete set of features (VGAE) and employing FS by ML algorithms voting (FS-VGAE). In both scenarios, the Early Stopping strategy defined operating points in early epochs, demonstrating quick adjustment of the encoder. It should be noted that, for the ELISA, the FS retained all variables; therefore, the FS-VGAE results are equivalent to the base model and are omitted from the previously mentioned table. The impact of FS was notable in more complex modalities. In ECHO, the FS-VGAE model achieved a reduction in validation error compared to the VGAE, decreasing from 2.117 to 0.102 in the chronic class. Similarly, in the Doppler modality, the FS-VGAE approach reduced the validation error in the acute class from 2.121 to 0.732.

**Table 1:** Convergence metrics and performance of VGAE models before and after feature selection (FS). Validation mean square error (MSE) is shown as Val MSE

Modality	Class	VGAE			FS-VGAE		
		Epoch	Train Loss	Val MSE	Epoch	Train Loss	Val MSE
ECG	Control	03	**1.253**	**0.321**	02	1.273	0.436
	Acute	08	1.511	0.590	13	**0.906**	**0.583**
	Chronic	22	0.819	**0.335**	13	**0.708**	0.373
ECHO	Control	11	0.527	0.373	05	**0.390**	**0.061**
	Acute	02	1.796	0.242	03	**1.731**	**0.081**
	Chronic	03	2.610	2.117	22	**0.383**	**0.102**
Doppler	Control	23	**0.938**	0.819	07	1.238	**0.813**
	Acute	08	**1.321**	2.121	02	2.321	**0.732**
	Chronic	04	**0.822**	**1.243**	03	1.852	1.314
ELISA	Control	40	0.028	0.003		**—**	
	Acute	15	0.301	0.033		**—**	
	Chronic	25	0.833	0.111		**—**	

*The best metrics achieved are shown in bold

Figure 5 displays the qualitative validation of the generated data by comparing the probability densities (KDE) of the original distribution (blue) with those produced by the VGAE model with all features (red) and the FS-VGAE model with FS by voting (green). Representative biomarkers were selected for visualization: ST Interval (ventricular repolarization) for ECG, Left Ventricle diameter at the end diastole (LVd) for ECHO, Ascending Aorta (AO) Pre-ejection time Average (Avg) for Doppler, and total IgG (IgGT) for ELISA.

### Classification of *T. cruzi* infection stages

The quantitative evaluation of the proposed approach was strictly aligned with the methodological protocol of Hevia-Montiel et al. [8] to ensure direct comparability of findings. The ACC (C1) and AUROC (C2) values obtained in the final evaluation are reported, dividing performance by infection stage and evaluating the impact of multimodal fusion versus baseline unimodal classifiers.

Table 2 compares the performance of the proposed GAT architecture with the reference algorithms (RF, ETC, DT, SVM) reported by Hevia-Montiel et al. [8]. The analysis is divided according to the classification task and the diagnostic mode.

**(a)** ***Control vs. acute:*** Graph modeling showed the greatest improvement in performance in each modality. In ECG, RF had an AUROC of *68.4%*, while GAT achieved an ACC of *83.3%* and an AUROC of *66.7%*. In terms of ECHO, the GAT model increased ACC values to *83.3%* and AUROC to *77.8%* in the test set, whereas the RF classifier achieved an ACC of *66.7%* and an AUROC of *69.3%*.

**(b)** ***Control vs. chronic:*** Serological markers (ELISA) reached performance metric limits of *100%* in all algorithms analyzed. In structural analysis (ECHO), ML algorithms showed an ACC of *50%*. In contrast, GAT achieved *83.3%* ACC and *77.8%* AUROC.

**(c)** ***Control vs. general infection:*** In this task, the GAT model performance in Doppler achieved an ACC of *66.7%* and AUROC of *55.6%*, while ML classifiers presented AUROC *<50%*. Furthermore, with ELISA, GAT increased the classification metrics to *100%*.

**Table 2:** Classification performance (%) in Final Evaluation for each modality

Classifier	ECG		ECHO		DOPPLER		ELISA	
	ACC	AUROC	ACC	AUROC	ACC	AUROC	ACC	AUROC
(a) Control vs. acute (N*=6*)								
RF	66.7	**68.4**	66.7	69.3	**66.7**	65.2	**100**	98.5
ETC	66.7	67.3	50.0	52.1	**66.7**	67.4	**100**	98.4
DT	50.0	53.2	66.7	65.2	33.3	40.3	**100**	97.3
SVM	50.0	52.4	50.0	54.4	**66.7**	**70.2**	83.3	90.7
GAT (Ours)	**83.3**	66.7	**83.3**	**77.8**	**66.7**	55.6	**100**	**100**
(b) Control vs. chronic (N*=6*)								
RF	**100**	**100**	50.0	66.7	**66.7**	**68.9**	**100**	**100**
ETC	**100**	**100**	50.0	58.9	**66.7**	67.5	**100**	**100**
DT	66.7	67.8	50.0	52.4	50.0	54.4	**100**	**100**
SVM	83.3	88.9	50.0	53.4	**66.7**	64.3	**100**	**100**
GAT (Ours)	83.3	**100**	**83.3**	**77.8**	**66.7**	55.6	**100**	**100**
(c) Control vs. general infection (N*=12*)								
RF	**83.3**	75.7	58.3	45.7	58.3	42.9	83.3	85.7
ETC	58.3	71.4	50.0	47.2	50.0	41.4	83.3	87.1
DT	50.0	51.4	50.0	51.4	50.0	41.7	83.3	82.9
SVM	50.0	52.3	50.0	51.3	50.0	45.7	80.6	85.7
GAT (Ours)	66.7	**77.8**	**66.7**	**100**	**66.7**	**55.6**	**100**	**100**

*The best metrics achieved are shown in bold

Below, Table 3 presents the comparison between our proposal and the algorithms reported by Hevia-Montiel et al. [8] in testing for each feature selection method.

**Table 3:** Multimodal classification performance (%) in Final Test with different feature selection strategies

Classifier	All features		Empirical selection		Voting selection	
	ACC	AUROC	ACC	AUROC	ACC	AUROC
(a) Control vs. acute (N*=6*)						
RF	83.3	85.8	**100**	**100**	83.3	92.3
ETC	83.3	94.3	83.3	89.4	83.3	93.5
DT	**100**	**100**	**100**	**100**	**100**	**100**
SVM	**100**	**100**	83.3	91.3	66.7	72.5
**GAT (Ours)**	83.3	66.7	66.7	66.7	83.3	**100**
(b) Control vs. chronic (N*=6*)						
RF	**100**	**100**	**100**	**100**	**100**	**100**
ETC	**100**	**100**	**100**	**100**	**100**	**100**
DT	**100**	**100**	**100**	**100**	**100**	**100**
SVM	66.7	75.4	83.3	91.4	**100**	**100**
GAT (Ours)	**100**	**100**	**100**	**100**	**100**	**100**
(c) Control vs. general infection (N*=12*)						
RF	**83.3**	82.9	83.3	85.7	83.3	90.0
ETC	**83.3**	84.3	75.0	88.6	75.0	94.3
DT	**83.3**	85.7	83.3	85.7	83.3	85.7
SVM	66.7	74.3	66.7	74.3	75.0	82.9
**GAT (Ours)**	66.7	**88.9**	**100**	**100**	**100**	**100**

*The best metrics achieved are shown in bold

**(a)** ***Control vs. acute:*** At this stage, using all features, the DT and SVM algorithms achieved ACC and AUROC of *100%*. For the ELISA modality, the GAT model achieved values of *83.3%* and *100%* for ACC and AUROC, respectively.

**(b)** ***Control vs. chronic:*** In the classification of the chronic stage, both the reference models and the GAT achieve *100%* performance for ACC and AUROC, respectively.

**(c)** ***Control vs. general infection:*** In this comparison, with the expert-selected empirical data, our model achieved *100%* performance. Similar results were obtained with voting selection, where our model achieved perfect classification on unseen data.

To mitigate the limitations of data scarcity, the impact of integrating synthetic data into the classification pipeline is evaluated. Performance achieved by augmenting the baseline GAT model via VGAE and FS-VGAE is detailed for unimodal and multimodal configurations. The effect of incorporating synthetic data into the training set of the classification models is quantified below in Table 4, which reports the results of cross-validation and final evaluations for each modality. The integration of VGAE acted as an effective regularization mechanism, reducing the generalization gap between cross-validation and final test, especially in high-dimensional modalities.

**Table 4:** Classification comparison (%) for each modality of GAT model with original data compared to those synthetically augmented by VGAE. Cross-validation results are expressed as mean and standard deviation. See panel A in Table 8 for additional classification metrics

Classifier	ECG		ECHO		DOPPLER		ELISA	
	ACC	AUROC	ACC	AUROC	ACC	AUROC	ACC	AUROC
(a) Control vs. acute: 5-fold cross-validation								
GAT	**73.3 ± 22.6**	66.7 ± 26.3	**73.3 ± 13.3**	86.7 ± 12.9	**63.3 ± 6.67**	**51.1 ± 11.3**	**86.7 ± 6.67**	**93.3 ± 13.3**
VGAE + GAT	66.7 ± 18.3	**68.9 ± 25.7**	70.0 ± 12.5	**91.1 ± 13.0**	53.3 ± 6.67	40.0 ± 11.3	**86.7 ± 6.67**	91.1 ± 13.0
(a) Control vs. acute: final test								
GAT	**83.3**	**66.7**	**83.3**	**77.8**	66.7	55.6	**100**	**100**
VGAE + GAT	**83.3**	**66.7**	**83.3**	**77.8**	**83.3**	**77.8**	**100**	**100**
(b) Control vs. chronic: 5-fold cross-validation								
GAT	**70.0 ± 12.5**	**71.1 ± 18.1**	**86.7 ± 6.67**	88.9 ± 9.94	**66.7 ± 10.5**	**80.0 ± 14.7**	**100 ± 0**	**100 ± 0**
VGAE + GAT	**70.0 ± 12.5**	**71.1 ± 18.1**	**86.7 ± 6.67**	**91.1 ± 8.31**	53.3 ± 6.67	58.9 ± 21.0	**100 ± 0**	**100 ± 0**
(b) Control vs. chronic: final test								
GAT	**83.3**	**100**	**83.3**	77.8	**66.7**	55.6	**100**	**100**
VGAE + GAT	**83.3**	**100**	**83.3**	**100**	50.0	**94.4**	**100**	**100**
(c) Control vs. general infection: 5-fold cross-validation								
GAT	66.7 ± 10.5	**62.2 ± 13.3**	**86.7 ± 12.5**	**93.3 ± 5.44**	**66.7 ± 10.5**	**60.0 ± 22.9**	**83.3 ± 14.9**	**84.4 ± 15.1**
VGAE + GAT	**73.3 ± 8.16**	60.0 ± 16.6	83.3 ± 10.5	**93.3 ± 5.44**	60.0 ± 8.16	60.0 ± 28.6	**83.3 ± 14.9**	**84.4 ± 15.1**
(c) Control vs. general infection: final test								
GAT	**66.7**	77.8	66.7	**100**	**66.7**	**55.6**	**100**	**100**
VGAE + GAT	50.0	**88.9**	**83.3**	**100**	**66.7**	44.4	**100**	**100**

*The best metrics achieved are shown in bold

**(a)** ***Control vs. acute:*** The Doppler modality showed the most significant improvement. The augmented version (VGAE + GAT) achieved a test AUROC of *77.8%*, while the base GAT model reached *55.6%*. In addition, ACC rose to *83.3%*.

**(b)** ***Control vs. chronic:*** Echocardiography achieved separability in the test set (ACC=AUROC=*100%*) after data augmentation. In the Doppler modality, the test AUROC increased to *94.4%* (compared to the baseline *55.6%*), with an ACC of *50.0%*.

**(c)** ***Control vs. general infection:*** The stability of the method was confirmed in the ELISA modality, where the metrics remained at saturation (*100%*) in the final test. In contrast, the ECHO modality improved the test accuracy from *66.7%* to *83.3%*.

Table 5 displays the impact of multimodal fusion, dimensionality reduction strategies, and data augmentation on classifier performance. The results confirm that voting selection is a robust configuration for the GAT model and that data augmentation with VGAE after FS is relevant for improving classification performance.

**Table 5:** Classification comparison (%) with different feature selection strategies of GAT model with original data compared to synthetically augmented data by VGAE and FS-VGAE. Cross-validation results are expressed as mean and standard deviation. See panel B in Table 8 for additional classification metrics

Classifier	All features		Empirical selection		Voting selection	
	ACC	AUROC	ACC	AUROC	ACC	AUROC
(a) Control vs. acute: 5-folds cross-validation						
GAT	**66.7 ± 10.5**	**66.7 ± 18.6**	**63.3 ± 12.5**	**62.2 ± 21.8**	60.0 ± 13.3	77.8 ± 19.9
VGAE + GAT	50.0 ± 0	48.9 ± 26.9	56.7 ± 17.0	55.6 ± 25.3	66.7 ± 14.9	77.8 ± 24.3
FS-VGAE + GAT	**—**	**—**	**—**	**—**	**80.0 ± 19.4**	**86.7 ± 13.0**
(a) Control vs. acute: final test						
GAT	**83.3**	**66.7**	66.7	66.7	83.3	**100**
VGAE + GAT	66.7	55.6	**83.3**	**100**	83.3	**100**
FS-VGAE + GAT	**—**	**—**	**—**	**—**	**100**	**100**
(b) Control vs. chronic: 5-folds cross-validation						
GAT	83.3 ± 10.5	95.6 ± 8.89	86.7 ± 12.5	93.3 ± 13.3	**96.7 ± 6.67**	**100 ± 0**
VGAE + GAT	**96.7 ± 6.67**	**97.8 ± 8.16**	**90.0 ± 8.16**	**93.3 ± 8.89**	90.0 ± 13.3	**100 ± 0**
FS-VGAE + GAT	**—**	**—**	**—**	**—**	**96.7 ± 6.67**	**100 ± 0**
(b) Control vs. chronic: final test						
GAT	**100**	**100**	**100**	**100**	**100**	**100**
VGAE + GAT	**100**	**100**	**100**	**100**	**100**	**100**
FS-VGAE + GAT	**—**	**—**	**—**	**—**	**100**	**100**
(c) Control vs. general infection: 5-folds cross-validation						
GAT	**73.3 ± 8.16**	**77.8 ± 21.1**	**80.0 ± 19.4**	68.9 ± 27.6	86.7 ± 12.5	93.3 ± 8.89
VGAE + GAT	70.0 ± 6.67	75.6 ± 14.7	**80.0 ± 19.4**	**86.7 ± 21.5**	86.7 ± 12.5	93.3 ± 8.89
FS-VGAE + GAT	**—**	**—**	**—**	**—**	**90.0 ± 8.16**	**95.6 ± 5.44**
(c) Control vs. general infection: final test						
GAT	66.7	**88.9**	**100**	**100**	**100**	**100**
VGAE + GAT	**83.3**	**100**	**100**	**100**	**100**	**100**
FS-VGAE + GAT	**—**	**—**	**—**	**—**	**100**	**100**

*The best metrics achieved are shown in bold

**(a)** ***Control vs. acute:*** In this stage, with the combination of voting selection and data augmentation (FS-VGAE+GAT), performance reached ACC=AUROC=*100%* in the final test. In the empirical selection strategy, the base GAT model had an AUROC in test of *66.7%*, while the addition of synthetic data increased discrimination to *100%*.

**(b)** ***Control vs. chronic:*** Metrics reached saturation (ACC=*100%*) in each evaluated scenario. As data increased, the cross-validation mean ACC improved from *83.3%* to *96.7%*.

**(c)** ***Control vs. general infection:*** During cross-validation with empirical selection, the mean AUROC increased from *68.9%* to *86.7%*. Furthermore, in the final test using all features, the augmented model had *16.6%* higher ACC than the base model (*83.3%* vs. *66.7%*). Finally, with voting selection, the classification reached *90.0%* and *95.6%* in validation, and *100%* in evaluation for ACC and AUROC, respectively.

### Interpretability: *Post Hoc* analysis

**Fig. 4:**
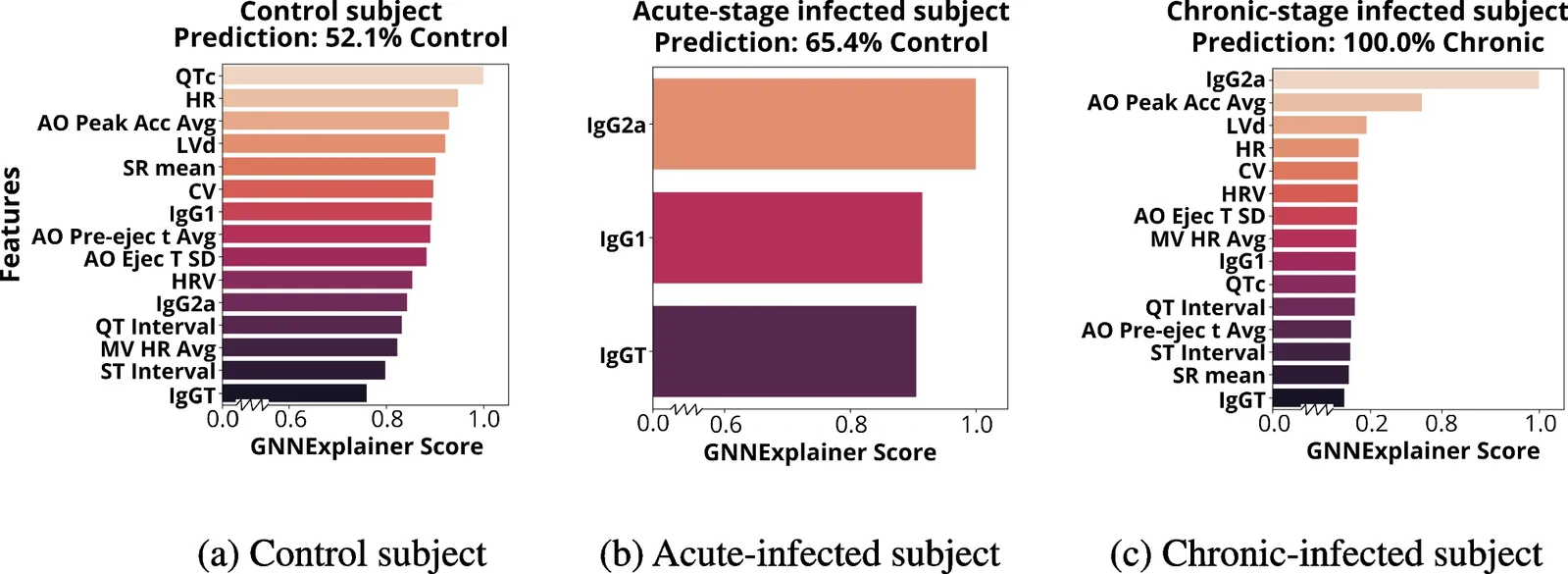
Interpretability analysis using feature importance according to GNNExplainer. The features shown are listed in Table 6

A supplementary interpretability study was carried out on representative subjects for a rigorous model validation, which allowed us to analyze the post hoc features’ importance by GNNExplainer. Three inference scenarios are shown in Fig. 4.

**(a)** ***Control subject*** (Fig. 4a): Multimodal analysis via VGAE+GAT model. The exemplified control subject was correctly classified with a marginal probability of *52%*. The GNNExplainer showed that QTc, Heart Rate (HR), AO Peak Acceleration Average, and LVd were the most relevant features for the final decision.

**b)** ***Acute-infected subject*** (Fig. 4b): Unimodal (ELISA) analysis via GAT model. The study with the sample subject using the GAT model encountered difficulties in detecting the acute stage (Probability of Acute: *35%*). The analysis shows a concentrated and diffuse distribution among the three serological markers.

**c)** ***Chronic-infected subject*** (Fig. 4c): Multimodal analysis via FS-VGAE+GAT model. During the classification of control vs. chronic, we observed that GNNExplainer identified IgG2a and AO Pre-ejection time Average as variables with great relevance in the final decision. The model classified correctly with high confidence (*100%*).

## Discussion

This section interprets the results in *T. cruzi* infection documented pathophysiology context and compares the performance of the proposed approach with the state-of-the-art methods reported by Hevia-Montiel et al. [8].

### Data augmentation

The convergence metrics reported in Table 1 demonstrated a quick adjustment of the encoder. The impact of Feature Selection was notably superior in complex modalities. The significant reduction in validation error in ECHO and Doppler modalities with FS-VGAE indicates that the removal of redundant variables facilitated the reconstruction of the latent topology and prioritized the structure of the most discriminating biomarkers over the noise inherent in the complete set.

**Fig. 5:**
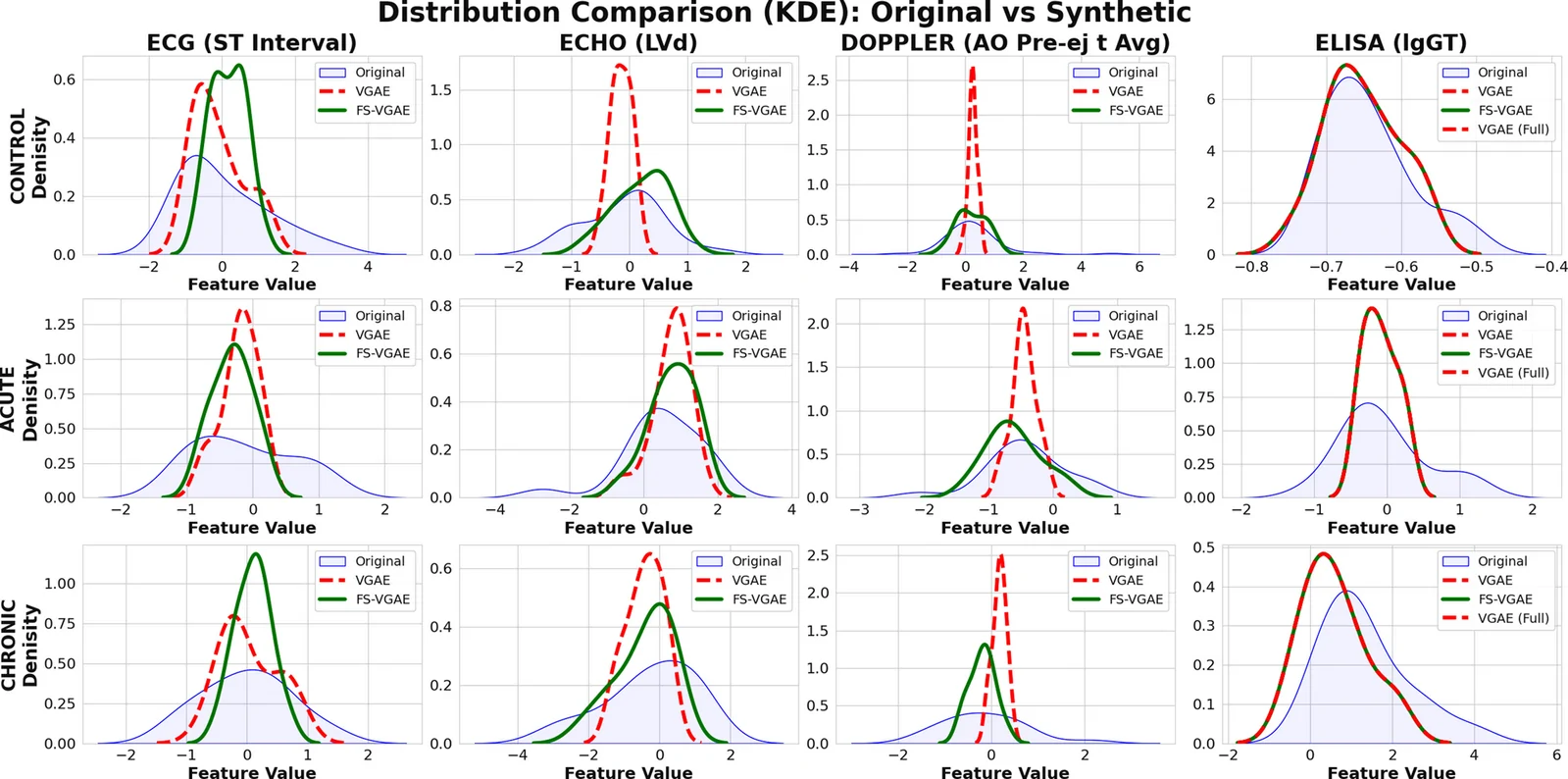
Comparison of probability densities (KDE) between original data and synthetic data generated by VGAE and FS-VGAE

Qualitatively (Fig. 5), it can be observed that the FS-VGAE approach tends to replicate the morphology of peaks and dispersion of real data in complex modalities more accurately, correcting the tendency of standard VGAE to adjust to the mean in high-dimensional spaces. This suggests that reducing input noise allows the generator to focus on relevant biological variability. In the case of ELISA, the overlap between VGAE and FS-VGAE curves is consistent with the methodology, confirming that both generative models converged to equivalent solutions when all characteristics are preserved. These results ensure that the synthetic data, especially after FS, respects the biological decision boundaries necessary for classification.

### Classification of *T. cruzi* infection stages

The proposed approach was strictly aligned with the methodological protocol of Hevia-Montiel et al. [8] to ensure direct comparability. In the control vs. acute comparison with unimodal information reported in Table 2, graph modeling showed the greatest improvement in performance. Although RF presented a slightly higher AUROC in ECG, GAT evidenced better generalization with a significantly higher ACC. In ECHO, the superiority of GAT (*16.6%* increase in good classification ratio compared to RF) indicates that the attention mechanism is effective in detecting initial changes in ventricular wall motion, patterns that linear or tree-based models cannot isolate with equal efficiency. For chronic-stage classification, while serological markers showed perfect linear separability, structural analysis (ECHO) revealed significant differences in robustness. The poor performance of ML algorithms (ACC *50%*) suggests inefficiency in discriminate between different classes. In contrast, the high performance of GAT confirms that fully connected graphs retain information about chronic cardiac development better than independent feature vectors. The GAT model has established itself as the leader in ECHO, Doppler, and ELISA modalities to detect general infection. The performance in Doppler is notable compared to the poor performance of ML classifiers (*AUROC <50%*). Additionally, GAT corrected classification errors detected in reference models for ELISA. The classification performance achieved is biologically expected from serological evaluations [1]; however, the dynamic topological weighting mechanism of the GAT maximizes the baseline discrimination, pushing the metrics to peak classification levels. These findings confirm the hypothesis that our GAT architecture, enhanced with ELU activation functions, effectively handles the diversity and scarcity of biomedical data, outperforming traditional ML methods.

Using multimodal FS strategies (Table 3), at first, we classified control vs. acute, where the lower performance of the GAT model compared to DT and SVM in this initial stage may be due to very slight acute pathophysiological changes and the small sample size, where traditional models are able to set stricter decision limits. However, GAT remained competitive in the ELISA modality. In long-term infection (chronic stage), the high and stable metrics across all models confirm that this stage shows a characteristic multimodal trace that can be easily separated. In the control vs. general infection comparison, the GAT model generally outperformed ML algorithms, improving on previously reported best models by over *10%* on each metric with empirical data and achieving perfect classification with voting selection. This demonstrates that our architecture, by building models of interactions between biomarkers rather than separate values, produced latent representations with a greater ability to generalize, capturing the infection’s pathophysiology, regardless of the specific stage.

The integration of VGAE acted as an effective regularization mechanism, reducing the generalization gap between cross-validation and final test, especially in high-dimensional modalities (Table 4). In control vs. acute, the improvement in Doppler indicates that the scattered areas of hemodynamic feature space were successfully populated by synthetic data, allowing the attention mechanism to establish stable decision boundaries. In control vs. chronic, ECHO achieved perfect separability after augmentation. However, the metric dissociation observed in Doppler (high AUROC, low ACC) indicates that the augmented model, by using a subset of high-dimensional features, did not fit well enough, causing values to cluster around the mean and adding noise in classification boundaries. Overall, our results show that the VGAE is helpful for modalities with complex nonlinear dependencies (such as ECHO and Doppler), while in linearly separable modalities (e.g., ELISA) it has a minor impact.

Regarding the multimodal classification (Table 5), the perfect performance achieved with FS-VGAE+GAT in the acute stage indicates that VGAE effectively compensates for the potential loss of information associated with manual expert selection. In the chronic stage, the improvement in cross-validation ACC demonstrates that it is essential to apply generative regularization to prevent overfitting when the model is faced with high-dimensional spaces. The results in general infection confirm that the VGAE+GAT architecture not only has the ability to handle multimodal heterogeneity but also reduces the dimensionality problem inherent in biomedical sources fusion.

It should be emphasized that the FS-VGAE+GAT proposal outperforms the baseline GAT model, and even the VGAE+GAT version, in each infection stage classification. This highlights the importance of FS in reducing the noise content that can be introduced in data augmentation.

### Interpretability: *Post Hoc* analysis

In Fig. 4a, the interpretability module suggests that, given the ambiguity of a borderline phenotype, due to its high importance in several features, the classifier prioritized electrophysiological stability (QTc) is the decisive factor in ruling out acute infection, although with low certainty due to its proximity to the decision boundary. The inability to prioritize a dominant feature in the GAT model led to a classification error, as shown in Fig. 4b for acute phase analysis. This indicates that in this particular subject, the antibody titers did not reach the discriminative threshold that the network had learned, acting as a profile similar to a control. In chronic stage (Fig. 4c), the behavior of the FS-VGAE+GAT model aligns with the expected pathophysiology of this phase, where the immune response is established (high antibody titers) and cardiac remodeling begins to impair ventricular ejection mechanics. The high confidence correct classification means that the network learned to effectively pair serological evidence of long-term infection with functional markers of hemodynamic deterioration, validating that the FS-VGAE strategy preserves and prioritizes the most clinically significant multimodal dependencies.

## Conclusion

This study demonstrates that Graph Attention Networks (GAT) are a robust tool for classifying experimental models with *T. cruzi* infection, even in a high dimensionality and limited data scenario. The integration of multimodal sources through fully connected graphs, driven by the generative regularization of VGAE, made it possible to recognize discrete nonlinear patterns that traditional machine learning algorithms fail to detect.

The superiority of our proposed FS-VGAE scheme stands out, demonstrating that refining the latent space through voting selection before synthesis is critical for reconstructing useful biological variability and stabilizing decision boundaries. As a result, perfect distinction (ACC=AUROC=*100%*) was achieved in the identification of the infection stages with multimodal fusion (FS-VGAE+GAT). The implementation of an interpretability scheme demonstrated that the high efficacy of the model is not a statistical artifact, but rather the product of learning consistent pathophysiological dependencies, such as the preference for mechanical dysfunction in acute stages and the high presence of antibodies in chronic phases. Conducting this study in an experimental model with *T. cruzi* infection allows underlying clinical analysis of biomarkers and effective modalities for Chagas disease detection, as well as examining the use of new study variables that are not currently used for this purpose in clinical scenarios. In this context, the heterogeneity of the analyzed data modalities (ECG, ECHO, DOOPLER, and ELISA), along with the biomarkers derived from each modality, has facilitated the study and supervised classification of the disease across different stages.

Current validation is limited to a controlled murine experimental environment, which requires careful consideration when extrapolating results directly to clinical practice in humans. Although VGAE demonstrated the ability to increase latent space density without introducing bias, reliance on a small baseline cohort (*N=72*) limits the capture of all the variability that exists in open populations with genetic diversity. These limitations will be taken into account in future research to ensure a good classification in real-world implementations. Nevertheless, our framework based on GAT and VGAE is adaptable. Its flexibility grants it strong generalization potential, allowing it to be readily translated to other multimodal medical domains and diagnostic challenges with high-dimensional and data-scarcity constraints. Furthermore, future work will explore expanding the current binary framework into a comprehensive multiclass classification model, enabling the simultaneous discrimination of all infection stages, alongside the integration of additional blood tests such as parasitological observation and Polymerase Chain Reaction (PCR).

## Data Availability

The data that support the findings of this study were available upon request from the corresponding author.

## Appendix A: Features by modality in the experimental murine model of this study

Table 6 summarizes the features (biomarkers) measured by clinical modality in the experimental murine model employed in this study for the *Trypanosoma cruzi* (*T. cruzi*) infection stage classification.

**Table 6:** Features by modality in the experimental murine model of infection by *T. cruzi* used in this study. Data adapted from Hevia-Montiel et al. [8]

Modality	ID Feature		
ECG	1. HR$^{+15,*8}$	6. R Interval	11. QTc$^{+11}$
	2. HRV$^{+13,*7}$	7. QRS	12. QTc dispersion
	3. CV$^{+14}$	8. QT Interval$^{+6,*9}$	13. SR mean$^{+4}$
	4. RR Interval	9. ST Interval$^{+9,*10}$	14. R Amplitude mean
	5. PQ Interval$^{*15}$	10. QTc Interval	
ECHO	15. HR SHORTH AXIS	17. LVs	19. EF$^{*5}$
	16. LVd$^{+5,*6}$	18. FS$^{*4}$	
Doppler	20. AbAO HR Avg	36. AO RR Interval Avg	51. AO Mean Acceleration Time SD
	21. AbAO HR SD	37. AO RR Interval SD	52. AO Peak Acceleration Avg$^{+7}$
	22. AbAO RR internal Avg	38. AO Pre-ejection time Avg$^{+8}$	53. AO Peak Acceleration SD
	23. AbAO RR interval SD	39. AO Pre-ejection time SD	54. MV HR Avg$^{+12}$
	24. AbAO Peak velocity Avg$^{*16}$	40. AO Peak velocity Avg$^{*11}$	55. MV HR SD
	25. AbAO Peak velocity SD	41. AO Peak velocity SD	56. MV RR Interval Avg
	26. AbAO Minimum Flow Velocity Avg	42. AO Stroke Distance Avg	57. MV RR Interval SD
	27. AbAO Minimum Velocity SD	43. AO Stroke Distance SD	58. MV E Peak velocity Avg$^{*13}$
	28. AbAO Mean Flow velocity Avg	44. AO Ejection time Avg$^{*12}$	59. MV E Peak velocity SD
	29. AbAO Mean Flow velocity SD	45. AO Ejection Time SD$^{+10}$	60. MV E Acceleration time Avg
	30. AbAO Pulsability Index Avg	46. AO Rise Time Avg$^{*14}$	61. MV E Acceleration time SD
	31. AbAO Pulsability Index SD	47. AO Rise Time SD	62. MV E Peak to Peak time SD
	32. AbAO Resistivity Index Avg	48. AO Mean velocity Avg	63. MV E Deceleration time SD
	33. AbAO Resistivity Index SD	49. AO Mean velocity SD	64. MV E Deceleration Rate SD
	34. AO HR Avg	50. AO Mean Acceleration Time Avg	
	35. AO HR SD		
ELISA	65. IgGT$^{+1,*1}$	66. IgG1$^{+3,*3}$	67. IgG2a$^{+2,*2}$

^+n^Voting variables selection.$^{*n}$Empirical variables selection. *n* indicates the relevance ranking

*Abbreviations:* **In ECG:** *HR* heart rate, *HRV* heart rate variability, *CV* cardiac variability, **In ECHO:** *LVd* left ventricle diameter at the end diastole, *LVs* left ventricle diameter at the end systole, *FS* fractional shortening, *EF* ejection fraction, **In Doppler:** *AO* ascending aorta, *AbAO* abdominal aorta, *MV* mitral valve, *Avg* Average, *SD* standard deviation, **In ELISA:** *IgGT* IgG total

## Appendix B: Training setups

Table 7 details the comprehensive training configurations, data splitting strategies, and the empirically determined hyperparameters employed for both the Variational Graph Autoencoder (VGAE) and the Graph Attention Network (GAT) models. The selected hyperparameter values were established through iterative empirical testing to optimize model convergence while mitigating overfitting within the constraints of the limited sample size.

**Table 7:** Training configurations and hyperparameters for the implemented generative (VGAE) and classification (GAT) models

Parameter / Configuration	VGAE	GAT
Data split strategy	Random split: (80%/20%)	5-fold stratified cross-validation: (66.6%/16.6%/16.6%)
Optimizer	Adam	Adam
Initial learning rate	$1 \times 10^{-3}$	$1 \times 10^{-4}$
Batch size (Graphs)	8	8
Maximum epochs	50	2000
Hidden layer units	1024	–
Latent space dimension (*z*)	16	–
Dropout rate	20%	–
L2 regularization (Weight decay)	–	0.01
Learning rate scheduler	Reduce on Plateau: (factor 0.5, patience 10)	Reduce on Plateau: (factor 0.5, patience 25)
Early stopping patience	10 epochs	200 - 300 epochs
Monitored validation metric	Mean Squared Error (MSE)	Cross-entropy loss

## Appendix C: Evaluation metrics

Classification performance was measured using ACC and AUROC. ACC is defined as the overall ratio of correct predictions made by the classifier:

C1 $$\begin{aligned} \text {ACC} = \frac{TP + TN}{TP + TN + FP + FN} \end{aligned}$$

where *TP* represents true positives, *TN* represents true negatives, *FP* represents false positives, and *FN* represents false negatives.

On the other hand, AUROC measures the model’s discrimination ability without taking into account the decision threshold. It is calculated as the definite integral from 0 to 1 of the ROC curve, which shows how the values of the true positive rate ($TPR = \frac{TP}{TP+FN}$) and the false positive rate ($FPR = \frac{FP}{FP+TN}$) relate to each other across various cutoff thresholds *τ *:

C2 $$\begin{aligned} \text {AUROC} = \int _{0}^{1} \text {TPR}(\tau ) \, d(\text {FPR}(\tau )) \end{aligned}$$

## Appendix D: VGAE: Probability densities (KDE)

Figure 5 compares the probability densities (KDE) of representative biomarkers, showing the overlap between the original dataset and the synthetic spaces produced by the VGAE and FS-VGAE approaches, to qualitatively assess the distribution of the generated data.

## Appendix E: Additional classification metrics

This appendix expands on the discriminative evaluation of the models by reporting Precision, Recall, and F1-Score in Table 8, alongside the ACC and AUROC metrics previously detailed (Tables 4 and 5).

**Table 8:** Classification comparison (%) in terms of supplementary metrics for the GAT model under different experimental setups: (A) evaluation by individual modality and (B) multimodal performance by feature selection strategies

(A) Performance by individual diagnostic modality												
Classifier	ECG			ECHO			DOPPLER			ELISA		
	Precision	Recall	F1-Score	Precision	Recall	F1-Score	Precision	Recall	F1-Score	Precision	Recall	F1-Score
(a) Control vs. acute: final test												
GAT	**87.5**	**83.5**	**83.0**	**87.5**	**83.5**	**83.0**	67.0	67.0	67.0	**100**	**100**	**100**
VGAE + GAT	**87.5**	**83.5**	**83.0**	**87.5**	**83.5**	**83.0**	**87.5**	**83.5**	**83.0**	**100**	**100**	**100**
(b) Control vs. chronic: final test												
GAT	**87.5**	**83.5**	**83.0**	**87.5**	**83.5**	**83.0**	**67.0**	**67.0**	**67.0**	**100**	**100**	**100**
VGAE + GAT	**87.5**	**83.5**	**83.0**	**87.5**	**83.5**	**83.0**	50.0	50.0	50.0	**100**	**100**	**100**
(c) Control vs. general infection: final test												
GAT	**67.0**	**67.0**	**67.0**	80.0	66.5	62.5	**80.0**	**66.5**	**62.5**	**100**	**100**	**100**
VGAE + GAT	50.0	50.0	50.0	**87.5**	**83.5**	**83.0**	**80.0**	**66.5**	**62.5**	**100**	**100**	**100**
(B) Multimodal performance by feature selection strategies												
Classifier	All features			Empirical selection			Voting selection					
	Precision	Recall	F1-Score	Precision	Recall	F1-Score	Precision	Recall	F1-Score			
(a) Control vs. acute: final test												
GAT	**87.5**	**83.5**	**83**	80.0	66.5	62.5	87.5	83.5	83.0			
VGAE + GAT	67.0	67.0	67.0	**87.5**	**83.5**	**83.0**	87.5	83.5	83.0			
FS-VGAE + GAT	**—**	**—**	**—**	**—**	**—**	**—**	**100**	**100**	**100**			
(b) Control vs. chronic: final test												
GAT	**100**	**100**	**100**	**100**	**100**	**100**	**100**	**100**	**100**			
VGAE + GAT	**100**	**100**	**100**	**100**	**100**	**100**	**100**	**100**	**100**			
FS-VGAE + GAT	**—**	**—**	**—**	**—**	**—**	**—**	**100**	**100**	**100**			
(c) Control vs. general infection: final test												
GAT	80.0	66.5	62.5	**100**	**100**	**100**	**100**	**100**	**100**			
VGAE + GAT	**87.5**	**83.5**	**83.0**	**100**	**100**	**100**	**100**	**100**	**100**			
FS-VGAE + GAT	**—**	**—**	**—**	**—**	**—**	**—**	**100**	**100**	**100**			

*The best metrics achieved are shown in bold

## Code Availability

The code used to generate the results is available at: https://github.com/gabrielcarcedo/Multimodal-GNN-for-Chagas-Classification.

## References

[1] Ascanio LC, Carroll S, Paniz-Mondolfi A, Ramírez JD (2024) In vitro diagnostic methods of chagas disease in the clinical laboratory: a scoping review. Front Microbiol 15:1393992 10.3389/fmicb.2024.1393992 PMC11091413 38746745

[2] Aho KA (2013) Foundational and Applied Statistics for Biologists Using R. Chapman and Hall/CRC, Boca Raton

[3] Brody S, Alon U, Yahav E (2022) How attentive are graph attention networks?. In: Proc ICLR

[4] Daniels LJ, Macindoe C, Koutsifeli P, Annandale M, James SL, Watson LE et al (2023) Myocardial deformation imaging by 2D speckle tracking echocardiography for assessment of diastolic dysfunction in murine cardiopathology. Sci Rep 13:12344. 10.1038/s41598-023-39446-w PMC10390581 37524893

[5] Garcia V, Bruna J (2017) Few-shot learning with graph neural networks. arXiv:1711.04043

[6] Haro P, Hevia-Montiel N, Perez-Gonzalez J (2023) Ecg marker evaluation for the machine-learning-based classification of acute and chronic phases of Trypanosoma cruzi infection in a murine model. Tropical Medicine and Infectious Disease 8:157. 10.3390/tropicalmed8030157 PMC10056492 36977158

[7] Higgins I, Matthey L, Pal A, Burgess C, Glorot X, Botvinick M, Mohamed S, Lerchner A (2017) beta-VAE: Learning basic visual concepts with a constrained variational framework. In: International conference on learning representations. https://openreview.net/forum?id=Sy2fzU9gl

[8] Hevia-Montiel N, Perez-Gonzalez J, Neme A, Haro P (2022) Machine learning-based feature selection and classification for the experimental diagnosis of Trypanosoma cruzi. Electronics 11:785. 10.3390/electronics11050785

[9] Hossain E, Nuzhat T, Masum S, Rahimi S, Golilarz NA (2026) R-GAT: cancer document classification leveraging graph-based residual network for scenarios with limited data: E. Hossain et al Scientific Reports 16(1):6582 10.1038/s41598-026-39894-6 PMC12913666 41703211

[10] Kingma D, Welling M (2014) Auto-encoding variational bayes. In: Proc ICLR

[11] Kipf T, Welling M (2016) Variational graph auto-encoders. arXiv:1611.07308

[12] Kipf T, Welling M (2017) Semi-supervised classification with graph convolutional networks. In: Proc ICLR

[13] Margeloiu A, Simidjievski N, Lio P, Jamnik M (2022) GCondNet: A novel method for improving neural networks on small high-dimensional tabular data. arXiv:2211.06302

[14] Pan American Health Organization (2025) World Chagas Disease Day 2025. https://www.paho.org/en/campaigns/world-chagas-disease-day-2025. Accessed 18 Feb 2026

[15] Rahmani AM, Yousefpoor E, Yousefpoor MS, Mehmood Z, Haider A, Hosseinzadeh M, Ali Naqvi R (2021) Machine learning (ML) in medicine: review, applications, and challenges. Mathematics 9:2970. 10.3390/math9222970

[16] Veličković P, Cucurull G, Casanova A, Romero A, Lio P, Bengio Y (2018) Graph attention networks. IN: Proc ICLR

[17] Vazquez B, Hevia-Montiel N, Perez-Gonzalez J, Haro P (2025) Weighted–VAE: A deep learning approach for multimodal data generation applied to experimental *T. cruzi* infection. PLOS ONE. 10.1371/journal.pone.0315843 PMC11932709 40127396

[18] World Health Organization (2025) Chagas disease (also known as American trypanosomiasis). https://www.who.int/news-room/fact-sheets/detail/chagas-disease-(american-trypanosomiasis). Accessed 18 Feb 2026

[19] Xuan P, Pan S, Zhang T, Liu Y, Sun H (2019) Graph convolutional network and convolutional neural network based method for predicting lncrna-disease associations. Cells 8:1012. 10.3390/cells8091012 PMC6769579 31480350

[20] Ying Z, Bourgeois D, You J, Zitnik M, Leskovec J (2019) GNNExplainer: Generating explanations for graph neural networks. Advances in Neural Information Processing Systems (NeurIPS). Curran Associates Inc, Red Hook, NY PMC7138248 32265580

